# How a brain keeps its cool

**DOI:** 10.7554/eLife.28109

**Published:** 2017-05-30

**Authors:** Swathi Yadlapalli, Orie T Shafer

**Affiliations:** Department of Molecular, Cellular and Developmental Biology, University of Michigan, Ann Arbor, United States; Department of Molecular, Cellular and Developmental Biology, University of Michigan, Ann Arbor, United Statesoshafer@umich.edu

**Keywords:** circadian rhythms, body temperature, temperature preference behavior, PDF neurons, DN2 neurons, thermosensory neurons, *D. melanogaster*

## Abstract

Temperature-sensing neurons in the *Drosophila* brain cooperate with the central circadian clock to help regulate body temperature.

**Related research article** Tang X, Roessingh S, Hayley SE, Chu ML, Tanaka NK, Wolfgang W, Song S, Stanewsky R, Hamada FN. 2017. The role of PDF neurons in setting preferred temperature before dawn in *Drosophila*. *eLife* **6**:e23206. doi: 10.7554/eLife.23206

Keeping body temperature within a certain range is essential for nearly all animals. Endothermic animals can generate their own heat and maintain a fixed body temperature, even when the temperature of their environment changes. Nevertheless, their core body temperature fluctuates modestly but predictably throughout the day, due to programmed changes in heat production and loss (Kräuchi and Wirz-Justice, 1994). In humans, for example, the core body temperature is lowest late at night (Aschoff, 1983). These daily changes are driven by a central circadian clock in the brain and they serve as important signals, both to synchronize other circadian clocks throughout the body (Buhr et al., 2010) and to ensure that we sleep at night (Kräuchi and Wirz-Justice, 2001).

Ectothermic animals, on the other hand, cannot produce their own heat, so their body temperature is determined by their environment. However, they have evolved different strategies to regulate their temperature. Iguanas, for example, vary their daily temperature by changing their level of activity throughout the day and also by moving around to take advantage of the different ambient temperatures within their environment (Tosini and Menaker, 1995). Thus, daily variations and rhythms in body temperature can be found in both endothermic and ectothermic animals, suggesting that they are a fundamental adaptation to life on a revolving planet.

Now, in eLife, Fumika Hamada and colleagues – including Xin Tang of Cincinnati Children’s Hospital Medical Center as first author – report new insights into the regulation of body temperature in the fruit fly *Drosophila melanogaster* (Tang et al., 2017). Previous work from the Hamada lab established that *D. melanogaster*, which is ectothermic, has a daily temperature preference rhythm. By switching its preference from higher temperatures during the day to lower temperatures at night, it produces daily body temperature rhythms similar to those seen in endothermic animals (Kaneko et al., 2012).

*Drosophila* has a relatively simple network of approximately 150 circadian clock neurons that are organized into distinct groups (Nitabach and Taghert, 2008). Each neuron has its own circadian clock, and together they form a network that serves as the master circadian pacemaker in the fly. The Hamada lab has previously shown that a small class of clock neurons, the dorsal neuron 2 group (or DN2s for short), regulate the temperature preference rhythm in the fly (Kaneko et al., 2012). Based on these findings, Tang et al. sought to identify the underlying neural pathways.

First, they revealed that a group of neurons called small ventral lateral clock neurons, which drive the fly’s pre-dawn bout of activity (Renn et al., 1999; Stoleru et al., 2004; Grima et al., 2004), are also required for setting the temperature preference in the hours preceding dawn. When these ventral lateral neurons were disrupted, the flies preferred abnormally low temperatures just before dawn. By contrast, when DN2s were disrupted, the flies preferred lower temperatures throughout their entire 24 hour-circadian cycle.

Tang et al. then examined whether these two groups of neurons are connected and, if so, how connections change during the course of the circadian cycle. They discovered that the projections of the ventral lateral neurons terminate next to those of the DN2s and that ventral lateral neurons excite the DN2s. The number of the neural connections between the two groups of neurons increased significantly throughout the night, peaking just before dawn and falling throughout the day. This suggests that the previously discovered morphological modifications of the ventral lateral neurons, such as the expansion and contraction of their axon terminals (Fernández et al., 2008), may cause the changes in thermal preference over the course of the day.

The researchers – who are based in Cincinnati, London and Japan – next addressed how external temperature and time of day are integrated in the brain to produce a temperature preference rhythm. This was guided by previous work, which had established that a group of neurons in the central brain called anterior cell neurons act as internal heat sensors (Hamada et al., 2008). Now, Tang et al. provide compelling evidence that these anterior cells modulate the ventral lateral neurons to regulate temperature preference before dawn; when the anterior cells were blocked, the fly’s preferred temperature was lowered. Thus, they have revealed a simple neural circuitry that drives the fly’s temperature preference rhythm. Within this network, the temperature-sensing anterior cells modulate ventral lateral neurons, which in turn activate the DN2s to elevate the preferred temperature before dawn.

The fly can use daily changes in the surrounding temperature as a cue to adjust its near 24-hour endogenous period and sleep-activity cycle to match the precise 24-hour light-dark cycle (Wheeler et al., 1993). Remarkably, the temperature-sensitive pathway implicated by Tang et al. does not appear to help adjust the central clock to daily temperature cycles. Thus, *Drosophila* seems to rely on distinct temperature-sensing pathways to produce and adjust to daily temperature rhythms.

By identifying the neural pathways underlying the daily regulation of temperature preference, Tang, Hamada and colleagues have revealed how the fly’s brain can generate a predictable daily body temperature rhythm – a remarkable feat for a such a small ‘thermoconformer’ that constantly has to face unpredictable temperature changes.
